# Multiplex Reverse-Transcription Loop-Mediated Isothermal Amplification Coupled with Cascade Invasive Reaction and Nanoparticle Hybridization for Subtyping of Influenza A Virus

**DOI:** 10.1038/srep44924

**Published:** 2017-03-21

**Authors:** Ying Chi, Yiyue Ge, Kangchen Zhao, Bingjie Zou, Bin Liu, Xian Qi, Qian Bian, Zhiyang Shi, Fengcai Zhu, Minghao Zhou, Lunbiao Cui, Chuan Su

**Affiliations:** 1 Department of Pathogen Biology, Key Laboratory of Pathogen Biology of Jiangsu Province, Nanjing Medical University, Nanjing, China; 2 Institute of Pathogenic Microbiology, Key Laboratories of Enteric Pathogenic Microbiology (Ministry of Health), Jiangsu Provincial Center for Disease Prevention and Control, Nanjing, China; 3 Department of Pharmacology, Jinling Hospital, Medical School of Nanjing University, Nanjing, China; 4 Department of Biomedical Engineering, Nanjing medical university, Nanjing, China

## Abstract

Considering the fatal human victims and economic loss caused by influenza virus infection every year, methodologies for rapid and on-site detection of influenza viruses are urgently needed. LAMP is the most commonly used nucleic acid isothermal amplification technology suitable for on-site use. However, for multiplex LAMP, differentiation of the amplicons derived from multiple targets is still challengeable currently. Here we developed a multiplex RT-LAMP assay for simultaneous amplification of three prominent subtypes of influenza viruses (A/H5, A/H7 and 2009A/H1). The amplicons were further identified by cascade invasive reaction and nanoparticle hybridization in separate target-specific detection tubes (referred to as mRT-LAMP-IRNH). The analytic sensitivities of the assay are 10 copies of RNA for all the three HA subtypes, and the specificity reached 100%. Clinical specimen analysis showed this assay had a combined sensitivity and specificity of 98.1% and 100%, respectively. Overall, the mRT-LAMP-IRNH assay can be used as a cost-saving method that utilizes a simple instrument to detect A/H5, A/H7, and 2009A/H1 influenza viruses, especially in resource-limited settings.

Influenza viruses are the major pathogens causing human respiratory diseases with severe morbidity and mortality worldwide^1^,^2^. Most influenza pandemics are associated with type A influenza viruses which can be further subdivided into subtypes based on the two types of viral surface glycoproteins, hemagglutinin (HA) and neuraminidase (NA)^3^. Among viruses isolated from aquatic birds, 16 HA and 9 NA subtypes have been identified to date^3^, although only a small subset (H1N1, H2N2, and H3N2) have acquired the capacity to cross species barriers and subsequently establish lineages in humans over the past century^4^. By antigenic drift and shift, influenza viruses evolve rapidly. The predominant seasonal influenza virus strain is 2009A/H1N1 in recent years, the cause of the 2009 H1N1 pandemic^5^,^6^. In addition, human infection of various subtypes of avian-origin influenza A viruses, especially H5 subtype (H5N1, H5N6) and H7 subtype (H7N7, H7N3, H7N9), has often been reported over the past few years^7^,^8^,^9^,^10^.

The rapid detection of these viruses is crucial for epidemiologic investigations and timely responses to an influenza pandemic threat. Molecular diagnostic methods based on nucleic acid amplification tests are more rapid and sensitive than traditional techniques including virus isolation and serological assays. Single or multiplex RT-PCR (including real-time RT-PCR) is at present the powerful method for detection of influenza viruses^11^,^12^,^13^,^14^,^15^. However, it requires bulky and expensive equipments, as well as highly skilled technicians, which make these methods not suitable for use in resource limited regions or for field use. Recently, researches have focused on the development of isothermal amplification methods for pathogen detection. As the reaction is conducted under isothermal conditions, it can be carried out with a simple water bath so that a thermal cycler is not required^16^. As the most commonly used isothermal amplification method at present, loop-mediated isothermal amplification (LAMP) method is rapid and sensitive for amplification of DNA at a constant temperature of 60–65 °C^17^,^18^. Besides targeting DNA templates, LAMP can also be used to amplify RNA template by the use of reverse transcriptase together with DNA polymerase, so-called reverse transcriptase LAMP (RT-LAMP). RT-LAMP methods have been developed to detect various RNA pathogens including influenza viruses^19^,^20^,^21^,^22^,^23^.

Simultaneous detection of multiple pathogens in one tube is without doubt cost- and time- saving. However, the differentiation of the ladder-like LAMP amplicons derived from multiple targets is still challengeable to date, although previous studies have described several methods for multiplex LAMP detection. These multiplex LAMP methods either used end point analysis, through gel electrophoresis^24^ or pyrosequencing^25^, or used real-time detection, through annealing curve analysis^26^, DARQ^27^ or MERT-LAMP technique^28^. However, these techniques all require complicated and specialized instrumentations, which diminish the point-of-care testing capability of multiplex LAMP.

Because the aggregation of gold nanoparticles (AuNPs) can cause the change of their optical property, AuNPs have been used as a sensor for DNA detection^29^,^30^,^31^. The main merit of this sensor is the visible detection by naked eyes, and thus especially suitable for field use. In this study, we developed a multiplex RT-LAMP assay for simultaneous amplification of three prominent subtypes of influenza A viruses (H5, H7 subtypes of avian influenza A and 2009A/H1N1 viruses), and the multiplex RT-LAMP amplicons were further identified by cascade invasive reaction^32^,^33^ and gold nanoparticle hybridization in separate target-specific detection tubes. Multiplex RT-LAMP coupled with cascade invasive reaction and nanoparticle hybridization (termed as mRT-LAMP-IRNH in brief) provides us a sensitive, specific and cost-saving diagnostic tool for identification of influenza A viruses, especially in resource-limited situations.

## Results

### Development and optimization of the mRT-LAMP-IRNH assay

**Figure 1:**
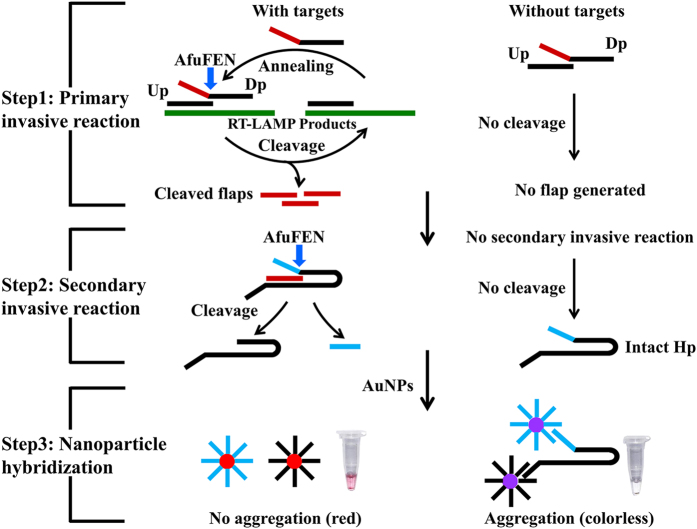
The principles of cascade invasive reaction and gold nanoparticle hybridization for detecting multiplex RT-LAMP products. Step 1 (Primary invasive reaction): A target DNA is firstly hybridized with an upstream probe (Up) and a downstream probe (Dp), forming a one-base overlapping structure at the 3′-end of the Up. The blue arrow indicates the site of cleavage. Step 2 (Secondary invasive reaction): The cleaved flaps from the target-specific primary invasive reaction are used to drive a secondary invasive reaction. Then the hairpin probe (Hp) is cleaved by AfuFEN. Step 3 (Nanopartical hybridization): When the target is present, the cleaved HP cannot trigger the aggregation of AuNPs, leading to a red color of the reaction. Conversely, when the target is absent, the Hp is intact, leading to the aggregation of AuNPs, and then the reaction becomes colorless.

At the commencement of this study, we used a real-time turbidimeter enabling observation of primer kinetics to determine the optimal primer sequences, primer concentrations, incubation temperature, as well as incubation time of the multiplex RT-LAMP reaction. The results showed that the optimal multiplex RT-LAMP reaction was obtained with primer sequences shown in Table 1 and the primer concentrations described in Materials and Methods. At 63 °C, the best amplification efficiency was observed for all the three HA subtypes (A/H5, A/H7 and 2009A/H1), and thus 63 °C was considered to be optimal temperature for the multiplex RT-LAMP reaction (data not shown). According to the amplification curves obtained by real-time turbidimeter, 50 min was selected as the optimal incubation time. In mRT-LAMP-IRNH, the multiplex RT-LAMP amplicons were further identified by cascade invasive reaction and nanoparticle hybridization in separate target-specific detection tubes. A schematic of the principles of cascade invasive reaction and gold nanoparticle hybridization is shown in Fig. 1. In the positive reaction, the cleaved hairpin probe cannot trigger the aggregation of AuNPs, leading to a red color of the reaction. Conversely, in the negative reaction, the intact hairpin probe leads to the aggregation of AuNPs, and then the reaction mixture becomes colorless (Fig. 1).

### Analytical sensitivity of the mRT-LAMP-IRNH

**Figure 2:**
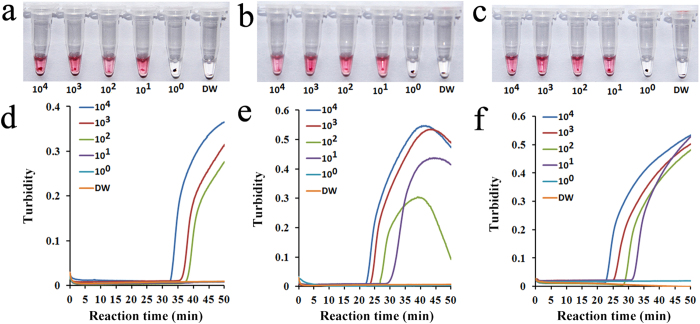
Analytical sensitivity of the mRT-LAMP-IRNH assay using serial dilutions of viral RNA transcripts as templates. A/H5 (**a**), A/H7 (**b**) or 2009A/H1 (**c**) *in vitro* RNA transcripts (ranging from 10^4^ to10^0^ RNA copies) were used as RT-LAMP template, respctively. The amplicons from each RT-LAMP reaction were further identified by cascade invasive reaction and gold nanoparticle hybridization in corresponding target-specific detection tube. The multiplex RT-LAMP amplification reactions for A/H5 (**d**), A/H7 (**e**), and 2009A/H1 (**f**) were also monitored by real-time turbidity detection and the corresponding curves of concentrations of templates were marked in the figure. DW: distilled water used as no-template control.

The analytical sensitivity of the mRT-LAMP-IRNH assay was determined by testing ten-fold serial dilutions of viral RNA transcripts (ranging from 10^4^ to10^0^ RNA copies) on two separate days. After 50 min of amplification, diluted RT-LAMP products were added into the invasive reaction mixture to perform cascade invasive reactions for each target, then the AuNPs were added for hybridization. The detection limits for all the three HA genes (A/H5, A/H7 and 2009A/H1) were 10^1^ copies of synthetic RNA by presenting red color in the reaction tubes as shown in Fig. 2a–c. Identical results were obtained on both days indicating that the mRT-LAMP-IRNH assay was both robust and reproducible.

To further evaluate the sensitivity of cascade invasive reaction and nanoparticle hybridization (IRNH) for LAMP product detection, the multiplex RT-LAMP amplifications were also monitored by a real-time turbidimeter. As shown in Fig. 2d–f, the detection limits for A/H7 and 2009A/H1 were both 10^1^ copies of synthetic RNA, which were equivalent to their detection limits obtained by mRT-LAMP-IRNH. However, the detection limit for A/H5 was 10^2^ copies of synthetic RNA, which was an order of magnitude lower than that obtained by mRT-LAMP-IRNH, indicating the sensitivity of cascade invasive reaction coupled with nanoparticle hybridization was higher than real-time turbidity detection for RT-LAMP product analysis.

### Analytical specificity of the mRT-LAMP-IRNH

**Figure 3:**
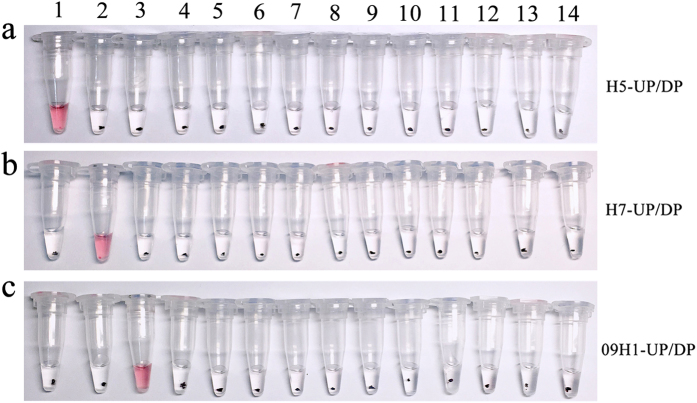
Specificity test results of the mRT-LAMP-IRNH assay for detection of influenza viruses. RNA extracted from influenza viruses A/H5N1, A/H7N9, 2009A/H1N1, or each control virus was used as RT-LAMP template, respctively. The amplicons from each RT-LAMP reaction were further identified by cascade invasive reaction and gold nanoparticle hybridization in three target-specific detection tubes. (**a**) Detection tube for A/H5. The cascade invasive reactions were performed with H5-UP, H5-DP and HP. (**b**) Detection tube for A/H7. The cascade invasive reactions were performed with H7-UP, H7-DP and HP. (**c**) Detection tube for 2009A/H1. The cascade invasive reactions were performed with 09H1-UP, 09H1-DP and HP. 1: A/H5N1; 2: A/H7N9; 3: 2009A/H1N1; 4: A/H1N1; 5: A/H3N2; 6: influenza B; 7: A/H9N2; 8: parainfluenza viruses types 1; 9: parainfluenza viruses types 2; 10: parainfluenza viruses types 3; 11: parainfluenza viruses types 4; 12: respiratory syncytial viruses types A; 13: respiratory syncytial viruses types B; 14: distilled water used as no-template control; UP: upstream probe; DP: downstream probe; HP: hairpin probe.

To assess the potential for the mRT-LAMP-IRNH assay to cross-react with other genetically or clinically related viruses which could cause similar symptoms, a specificity test was conducted using viral RNA extracts from various control viruses. As shown in Fig. 3, positive reactions with red color were only observed in the preparations of A/H5N1, A/H7N9 and 2009A/H1N1 viruses, whereas none of the control viruses showed a positive result, indicating the high specificity of the mRT-LAMP-IRNH assay.

### Clinical sample analysis by the mRT-LAMP-IRNH

To evaluate the performance characteristics of the mRT-LAMP-IRNH assay in clinical sample detection, a total of 88 clinical specimens were subjected to mRT-LAMP-IRNH assay with the parallel analysis by real-time RT-PCR. The results showed that, out of 52 specimens that were positive for influenza as detected by real-time RT-PCR, mRT-LAMP-IRNH detected 6/6A/H5, 18/18A/H7 and 27/28 2009A/H1. The sensitivity and specificity for detecting A/H5 were 100% (6/6) and 100% (82/82), respectively. For detecting A/H7, the sensitivity and specificity were also 100% (18/18) and 100% (70/70) respectively, and for 2009A/H1, the sensitivity and specificity were 96.4% (27/28) and 100% (60/60), respectively. Based on combined A/H5, A/H7 and 2009A/H1 detection, the mRT-LAMP-IRNH assay had an overall sensitivity and specificity of 98.1% (51/52) and 100% (36/36), respectively (Table 2).

## Discussion

Viral culture paired with serological HA typing is the standard method for detecting and typing influenza A viruses^34^. The main drawbacks of virus culture are that it can only detect live viruses and requires more time and higher biosecurity. Immunological methods for testing influenza viruses have low skill requirements, but poor sensitivity and specificity. Recently, many molecular diagnostic approaches such as single or multiplex RT-PCR (including real-time RT-PCR) have been developed for subtyping influenza viruses. However, these methods require expensive thermal cycling equipments. Currently, rapid, reliable and affordable point-of-care tests for influenza virus detection are urgently needed, especially in resource limited regions. In this study, we describe a sensitive mRT-LAMP-IRNH assay for the detection of three influenza A viruses (subtypes A/H5, A/H7 and 2009A/H1) for the first time. The read-out can be observed by naked eyes, and no specialized instrument is required, which make this assay especially useful in resource-limited situations such as primary care facilities.

As LAMP belongs to isothermal amplification methods, it can be carried out with a simple water bath and without the need of bulky and expensive equipments, which points to the potential applicability of the assay for clinical point-of-care diagnostic use. LAMP products can be detected by agarose gel electrophoresis, turbidity or fluorescence detection^35^,^36^, lateral flow dipstick^23^, or even visual inspection^37^,^38^. These methods are robust and reliable, but detect total amplification products in a reaction and are thus limited to detection of a single target. Multiplex detection is the development trend of pathogen detection technology due to the properties of cost savings and high efficiency. In order to achieve multiplex LAMP detection, methods based on end point analysis^24^,^25^ as well as real-time detection^26^,^27^,^28^ have been employed to differentiate multiple target sequences, while these strategies all required complicated and specialized instruments. To enable multiplex pathogen detection without the need of specialized instruments, Dou *et al*. developed a polymer/paper hybrid microfluidic biochip for simultaneous LAMP detection of three meningitis-causing pathogens. Though this assay was not really a multiplex LAMP reaction, by using of microfluidic biochip, three pathogens were simultaneously detected on a chip, and high sensitivity and specificity were achieved within 1 h^39^. In this study, we present an alternative molecular method for multiplex LAMP amplicon detection by combining invader techniques^32^,^33^ and gold nanoparticle probe techniques^29^,^30^,^31^. AuNPs have been used as a sensor for DNA detection for years. To improve the sensitivity, we coupled invader techniques with gold nanoparticle probe techniques, which were further used to detect multiplex RT-LAMP amplicons for the first time. Although the identification of the multiplex RT-LAMP amplicons is monoplex, the mRT-LAMP-IRNH assay developed in this study is a cost-saving method that requires no complicated instrument, and is more suitable for multiple-target detection of limited amount and/or precious nucleic acids. This pilot study also provides a strategy for establishing multiplex LAMP assay much more than three plex for field use. Meanwhile, unknown clinical specimens with co-infection can also be distinguished without using any equipments.

Due to the use of six sequence-specific primers per target, similar to that of the ordinary LAMP technique, which recognizes eight conserved regions for specific identification of positive targets, mRT-LAMP-IRNH offers the same high specificity. Moreover, the invasive reaction was a specific DNA detection method by the use of two target-specific probes. The mRT-LAMP-IRNH assay combines the specificities of both LAMP and the cascade invasive reaction, which should provide higher specificity in theory. In this study, we demonstrated the high specificity of the assay by showing the absence of cross-reactivity by analyzing the RNA extracts from various genetically or clinically related viruses which could cause similar symptoms.

The concordance of high analytical sensitivity between LAMP and other sensitive molecular methods has been reported previously^40^,^41^. LAMP reaction was able to tolerate the inhibitory effect of large amounts of templates, and was less affected by the presence of various salts and inhibitors^42^. In mRT-LAMP-IRNH method, aggregation of AuNPs is achieved by adding hairpin probes complementary to both AuNPs. The cleavage of hairpin probes is triggered by the cleaved flaps from the target-specific primary invasive reaction. Because one LAMP amplicon can yield large numbers of cleaved hairpin probes, the sensitivity of this method is increased significantly. By testing ten-fold serial dilutions of viral RNA transcripts, the analytical sensitivities of the mRT-LAMP-IRNH assay were found to be 10^1^ copies of synthetic RNA for all three targets. Previous studies have shown that although multiplex nucleic acid detection tests have the advantage of high efficiency, due to the mutual interference of multiple primers, the analytic sensitivities of these assays tend to decrease. However, through the signal amplification effects of invader techniques and gold nanoparticle probe techniques, the sensitivity of the mRT-LAMP-IRNH assay established in this study was equivalent or even significantly increased as compared to general monoplex LAMP method^23^,^43^,^44^,^45^. In this study, the detection limit for A/H5 obtained by mRT-LAMP-IRNH was ten times higher than that obtained by real-time turbidity detection, indicating the cascade invasive reaction coupled with AuNPs hybridization increased the sensitivity of LAMP whose results were usually analyzed through turbidity.

The performance characteristics of the mRT-LAMP-IRNH assay were evaluated by testing 88 clinical specimens with the parallel analysis by real-time RT-PCR. Compared to real-time RT-PCR, the sensitivities of the mRT-LAMP-IRNH assay for detecting A/H5, A/H7 and 2009A/H1 were 100%, 100% and 96.4%, respectively. As 50 min was selected for multiplex LAMP reaction, the total detection time of mRT-LAMP-IRNH assay was about 100~120 min which was similar to that of real-time RT-PCR assay. As 120 min might be a little long for field use, we had tried to shorten the invasive reaction and hybridization reaction time, but we found that when the concentration of virus was low, the result might be somewhat ambiguous to the naked eye if the detection time was shortened. In future, we would test more conditions and settings to try to shorten the reaction time, and to combine invasive reaction with hybridization reaction in one step. These would make the assay more timely and practical for field use.

In summary, a highly sensitive and specific mRT-LAMP-IRNH assay was developed for the first time in this study which can detect three prominent subtypes of influenza viruses (A/H5, A/H7 and 2009A/H1). The advantages of cost-saving and no requirement of any complicated instrument make this assay more suitable for low-equipment setting laboratory use and for on-site testing. Consequently, this detection method constructed in this study would facilitate initial clinical treatment, infection control, as well as epidemiologic investigations of influenza infection.

## Methods

### Ethics statement

Written informed consent for using the clinical specimens was obtained from all patients involved in this study. This research was approved by the Ethics Committee of the Jiangsu Provincial Center for Disease Prevention and Control. The methods were carried out in accordance with the Ethical Guidelines for Medical and Health Research Involving Human Subjects.

### Viral isolates and clinical specimens

Influenza virus strains (A/Nanjing/2/2013 (H7N9), A/Jiangsu/1/2007(H5N1), and A/Jiangsu/2/2009(H1N1)) isolated from patient samples at Jiangsu Provincial Center for Disease Prevention and Control were included in the mRT-LAMP-IRNH assay development. Other genetically or clinically related virus isolates including seasonal influenza viruses (A/H1N1, A/H3N2, and B), avian influenza virus A/H9N2, parainfluenza viruses (types 1, 2, 3, and 4), and respiratory syncytial viruses (types A and B) were used as control viruses to assess the specificity of the assay. For methodological evaluation of this study, a total of 84 nasopharyngeal swabs were collected from influenza-like cases. Among these specimens, 48 had been identified positive for influenza A viruses including 18A/H7N9, 2A/H5N1, and 28 2009A/H1N1, and 36 had been identified negative for influenza A viruses. Furthermore, 4 known positive poultry cage surface swabs (2A/H5N1 and 2A/H5N6) were collected from live poultry markets. All these isolates and specimens were stored at −80 °C until use. This study was approved by the Ethics Committee of Jiangsu Provincial Center for Disease Prevention and Control.

### Extraction of viral RNA

Viral RNA was extracted from 200 μl of samples with a MagNA Pure LC Total Nucleic Acid Isolation Kit on a MagNA Pure^TM^ system (Roche Diagnostics, Manheim, Germany) according to the manufacturer’s instructions. The extracted RNA was dissolved in 50 μl elution buffer (*pH* = 8.0) and kept at −80 °C until use.

### Preparation of transcripts

The HA genes were amplified from three influenza virus strains (A/Nanjing/2/2013(H7N9), A/Jiangsu/1/2007(H5N1), and A/Jiangsu/2/2009(H1N1)) with primers containing T7 promoter sequence in the reverse sides. PCR amplicons containing the full length region of each gene target for multiplex RT-LAMP were *in vitro* transcribed with T7 RNA polymerase (TaKaRa Biotechnology Co. Ltd., Dalian, China) according to the manufacturer’s instructions. The synthetic RNA transcripts were purified, quantified, and then tenfold diluted ranging from 10^4^ to10^0^ RNA copies/μl.

### Design of primers and probes

Multiplex RT-LAMP primers for avian influenza A/H5, A/H7 and 2009A/H1 were designed using conserved regions of the HA gene for each influenza A subtype, respectively. Primers were designed using the PrimerExplorer version 4 program (Eiken Chemical Co., Tokyo, Japan). The feasibility and specificity of the primers were subsequently checked by BLAST search with sequences in GenBank. The sequence information of the final selected multiplex RT-LAMP primers is shown in Table 1. The cascade invasive reaction upstream probes and downstream probes were designed using the Universal Invader Design Software version 1.2.4 (Third Wave Technologies, Inc., Madison, USA) according to the sequences of multiplex RT-LAMP amplification products. The sequence information of the final selected upstream probes, downstream probes, hairpin probes used in secondary invasive reaction, as well as the two probes used for modification of AuNPs is shown in Table 3. All the primers and probes were synthesized by TaKaRa Biotechnology Co. Ltd. (Dalian, China).

### Preparation and modification of AuNPs

AuNPs were synthesized by reducing tetrachloroauric acid with trisodium citrate according to the protocol described previously^46^. Transmission electron micrographs were taken to measure the size of synthetic particles (an average diameter of 13 nm). Two types of oligonucleotide probes (Table 3) were used to separately modify the two batches of the AuNPs.

### Multiplex RT-LAMP reaction

Multiplex RT-LAMP reaction was carried out with a RNA Amplification Kit (RT-LAMP) (Eiken China Co., Ltd., Shanghai, China). The reaction was performed in 20 μl of a mixture containing 10 μl of Reaction Mix, 0.8 μl of Enzyme Mix, 18 primers for avian influenza A/H5, A/H7and 2009A/H1 (each of the outer primers F3 and B3 at 0.15 μM, each of the inner primers FIP and BIP at 1.2 μM, and each of the loop primers LF and LB at 0.6 μM final concentration), and 1 μl of RNA template. The reaction mixture containing 1 μl of distilled water was used as no-template controls. Multiplex RT-LAMP amplification reactions were carried out at 60, 63, and 65 °C to determine the shortest amplification time and best detection performance. During assay development, including sensitivity and specificity tests, a real-time turbidimeter (LA320C, Teramecs, Tokyo, Japan) was used for RT-LAMP amplification reaction. And a heat-block (ThermoStat Plus, Eppendorf, Hamburg, Germany) was used in the analysis of clinical samples.

### Cascade invasive reaction and gold nanoparticle hybridization

For each multiplex RT-LAMP reaction, three target-specific cascade invasive reactions were performed. The 20 μl invasive reaction was carried out with 0.2 μM upstream probe, 0.2 μM downstream probe, 0.2 μM hairpin probe, 100 ng AfuFEN enzyme which was prepared in our lab as described before^47^, and 5 μl diluted multiplex RT-LAMP amplification products (20 μl products plus 30 μl deionized water) in a reaction buffer containing 10 mM MOPS (pH7.5), 0.05% Tween-20, 0.05% Nonidet P40, and 7.5 mM MgCl_2_. The reactions were run at 85 °C for 1 min followed by 63 °C for 20 min. After cascade invasive reaction, 3 μl of each AuNPs (30 nM), NaCl (500 mM) and water were added in a volume of 30 μl. Hybridization was performed at 55 °C for 30 min. The results were observed by naked eyes directly or after briefly centrifuging the products. The positive reaction mixture kept red while the negative reaction mixture became colorless.

### Sensitivity and specificity of mRT-LAMP-IRNH

Ten-fold serial dilutions of synthetic RNA transcripts of the three HA genes (avian influenza A/H5, A/H7 and 2009A/H1, ranging from 10^4^ to10^0^ RNA copies) were used to assess the analytical sensitivity of the mRT-LAMP-IRNH assay. The specificity of the assay was determined by analyzing the RNA extracts from various control viruses mentioned above. Briefly, RNA extracted from influenza viruses A/Nanjing/2/2013(H7N9), A/Jiangsu/1/2007(H5N1), A/Jiangsu/2/2009(H1N1), or each control virus was used as RT-LAMP template, respectively. The amplicons from each RT-LAMP reaction were further identified by cascade invasive reaction and gold nanoparticle hybridization as described before in three separate target-specific detection tubes.

### Clinical specimen analysis

To investigate the feasibility of our methodology for clinical sample analysis, a total of 88 clinical specimens were collected and analyzed by mRT-LAMP-IRNH assay with the parallel analysis by our in-house real-time RT-PCR assays for influenza A/H5, A/H7 and 2009A/H1. The primers and probes used in our real-time RT-PCR assays for A/H5, A/H7 and 2009A/H1 were all recommended by WHO^48^,^49^,^50^, and all the three assays had been validated against viral culture and commercial real-time RT-PCR kits. The Real-time RT-PCR was performed using a SuperScript^®^ III Platinum One-Step qRT-PCR System (Invitrogen) according to the instructions.

## Additional Information

**How to cite this article:** Chi, Y. *et al*. Multiplex Reverse-Transcription Loop-Mediated Isothermal Amplification Coupled with Cascade Invasive Reaction and Nanoparticle Hybridization for Subtyping of Influenza A Virus. *Sci. Rep.* **7**, 44924; doi: 10.1038/srep44924 (2017).

**Publisher's note:** Springer Nature remains neutral with regard to jurisdictional claims in published maps and institutional affiliations.

## Figures and Tables

**Table 1:** Primers used for multiplex RT-LAMP assay.

Target	Name	Sequence (5′-3′)	Length (mer)
A/H5	H5-F3	GCTATAGCAGGKTTTATAGAGG	22
	H5-B3	GCCTCAAACTGAGTGTTCAT	20
	H5-FIB	ACTCCCCTGCTCRTTGCTATGGATGGCAGGGAATGGTA	38
	H5-BIP	GGTACGCTGCAGACAARGAATGAGTTGACCTTATTGGTGA	40
	H5-LF	GGTGRWACCCATACCAACCA	20
	H5-LB	CYACTCAAAAGGCAATAGATGGA	23
A/H7	H7-F3	TGTCTGTTATCCTGGGAAAT	20
	H7-B3	AGCATTATCTGTGTTTGACAG	21
	H7-FIB	GTATGTGAATCCCATTGCTTCCTTGGTGAATGAAGAAGCTCTGAGG	46
	H7-BIP	GGAATAAGAACTAATGGARCAACCAAGCCATTTCATTTCTGCATAG	46
	H7-LF	CCGCCTGATTCTCTGAGWATTT	22
	H7-LB	GCATGTAGGAGATCAGGATCTTCA	24
2009A/H1	09H1-F3	CCGGGAGACAAAATAACATTC	21
	09H1-B3	GTATATTCTGAAATGGGAGGC	21
	09H1-FIB	CAGATCCAGCATTTCTTTCCATTGGAAGCAACTGGAAATCTAGTG	45
	09H1-BIP	TATCATTTCAGATACACCAGTCCACTGGTGTTTATAGCACCCTTG	45
	09H1-LF	CGAATGCATATCTCGGYAC	19
	09H1-LB	ATACAACTTGTCARACACC	19

**Table 2:** Performance of mRT-LAMP-IRNH compared with real-time RT-PCR for detecting influenza A/H5, A/H7 and 2009A/H1.

Target	mRT-LAMP-IRNH^a^		Performance characteristics	
	Positive	Negative	Sensitivity	Specificity
**A/H5**	6/6	82/82	100%	100%
**A/H7**	18/18	70/70	100%	100%
**2009A/H1**	27/28	60/60	96.4%	100%
**Combined A/H5, A/H7 and 2009A/H1**	51/52	36/36	98.1%	100%

^a^The results of real-time RT-PCR were used as the reference standard.

**Table 3:** Probes used for cascade invasive reaction and modification of AuNPs.

Name	Type	Sequence (5′-3′)	Modification
H5-UP	upstream probe	TCTTTGTCTGCAGCGTACCCT	
H5-DP	downstream probe	CGCGCCGAGG ACTCCCCTGCTCATT	
H7-UP	upstream probe	TCCATTAGTTCTTATTCCACTGTATGTGAAC	
H7-DP	downstream probe	CGCGCCGAGG TCCCATTGCTTCCTTG	
09H1-UP	upstream probe	ATCTGGTATTATCATTTCAGATACACCAGTT	
09H1-DP	downstream probe	CGCGCCGAGG CCACGATTGCAATACAAC	
HP	hairpin probe	GTCTTGTGGTACTGCACTCGTCTCGGTTTTCCGAGACGAGTCCTCGGCGCGATCGTGATGAACCAT	
3′-AuNP	nanoparticle probe	GCAGTACCACAAGACAAAAAAAAAA	3′-SH C6
5′-AuNP	nanoparticle probe	AAAAAAAAAAATGGTTCATCACGAT	5′-SH C6
